# OBJECTIVE AND SUBJECTIVE EVALUATION OF ADOLESCENT’S ORTHODONTIC TREATMENT NEEDS AND THEIR IMPACT ON SELF-ESTEEM

**DOI:** 10.1590/1984-0462/;2017;35;1;00003

**Published:** 2017

**Authors:** Anshika Sharma, Anmol Mathur, Manu Batra, Diljot Kaur Makkar, Vikram Pal Aggarwal, Nikita Goyal, Puneet Kaur

**Affiliations:** a Departamento de Ortodontia em Saúde Pública, Surendera Dental College and Research Institute, Sri Ganganagar, Rajastão, Índia.

**Keywords:** adolescent, index of orthodontic treatment need, aesthetics, malocclusion

## Abstract

**Objective:**

To investigate the presence of association between objective and subjective evaluation of orthodontic treatment needs in adolescents and their impact on their self-esteem.

**Methods:**

Cross-sectional study with adolescents aged 10-17 years old in Sri Ganganagar city, Rajasthan, India. The objective index of orthodontic treatment need (IOTN) dental health component (DHC) and the subjective aesthetic component (AC) were used to determine the normative and the self-perception need for orthodontic treatment, respectively. The selected students were further examined for dental trauma, tooth loss, and dental caries. Rosenberg Self-Esteem Scale was applied for self-esteem level determination. Linear regression analysis was executed to test the individual association of different independent clinical variables with self-esteem scores.

**Results:**

Among 1,140 studied adolescents, the prevalence of dental normative orthodontic treatment need was in 56.9% of individuals, whereas 53.3% of individuals considered themselves as needy for the treatment. Multivariate analyses revealed that out of all dental disorders, DHC followed by AC of IOTN had maximum impact on the self-esteem of the adolescence.

**Conclusions:**

Dissatisfaction with dental appearance is a strong predictor for low self-esteem in adolescence.

## RESUMO

**Objetivo:**

Verificar a presença de associação entre a avaliação objetiva e subjetiva da necessidade de tratamento ortodôntico em adolescentes e seu impacto na autoestima.

**Métodos:**

Estudo transversal com adolescentes de 10 a 17 anos da cidade de Sri Ganganagar, Rajastão, Índia. Foi utilizado o Índice de Ortodontia de Saúde Dental (IOTN) para avaliar de modo objetivo a necessidade de tratamento (DHC) e o componente estético desse índice (AC) para a avaliação subjetiva. Os estudantes selecionados foram também avaliados quanto a traumatismos dentários, perda dentária e cárie dentária. Aplicou-se a escala de autoestima de Rosenberg para determinar o nível de autoestima dos estudantes. Os dados foram analisados por regressão linear múltipla, testando a associação individual das diferentes variáveis clínicas independentemente da autoestima dos adolescentes, de acordo com o escore da escala de Rosenberg.

**Resultados:**

Entre 1.140 adolescentes estudados, a prevalência da necessidade de tratamento ortodôntico avaliado de modo objetivo pelo IOTN-DHC ocorreu em 56,9% dos indivíduos. A avaliação subjetiva do adolescente (IOTN-AC) revelou prevalência de necessidade de tratamento de 53,3%. A análise multivariada mostrou que, além de todos os problemas dentários, o componente objetivo (IOTN-DHC), seguido do componente estético subjetivo (IOTN-AC), teve maior impacto na autoestima dos estudantes analisados.

**Conclusões:**

A insatisfação com a aparência dental é um forte preditor de baixa autoestima na adolescência.

## INTRODUCTION

One-fifth of the world’s population is adolescent. This term is defined as a person between 10 and 19 years old by the United Nations Children’s Fund (UNICEF).^1^ In the adolescence stage, individuals undergo extensive physical, psychological, emotional, and personality transformations, which determine an identity that will influence their future.^2^ During this period, individuals are extremely concerned with their appearance and are particularly in a need to improve their appearance and social attractiveness. Facial attractiveness plays a major role in the socialization, and adolescents with a normal dental appearance are often judged to be better-looking and more desirable among friends.^3^

A smile is one of the most effective measures by which people convey their emotions and, moreover, a beautiful smile is an added asset to a beautiful face.^4^ As adolescents are very sensitive about their facial aesthetics, malocclusion can often lead to a conscious effort to hide or avoid their smile, thus lowering their self-confidence. It is assumed that having a harmonious smile may increase levels of self-esteem in adolescents and, hence, their ability to interact appropriately in society.^5^

Malocclusion is the second most prevalent oral pathology,^6^ which not only affects physical appearance of an individual, but also has other consequences. Individuals with malocclusion, for instance, can develop feeling of embarrassment about their dental arrangement and feel shy in social contacts. This might affect their self-esteem, that is a socially derived construct and a product of many factors, such as confidence, self-image, self-awareness, self-respect, attitudes, values, and self-worth.^7^ It has a strong relation to happiness, and the presence of low self-esteem is likely to lead toward depression under some circumstances.^8^

Self-esteem or one’s overall evaluation or appraisal of one’s own value is associated with greater life satisfaction and fewer health problems.^9^ The social background influence and self-perception are important factors which play a vital role on a person’s self-esteem toward malocclusion. Some patients with severe malocclusion are satisfied or indifferent about their aesthetics, while others with minor irregularities are very much concerned about their aesthetics. That is, the normative and subjective need of the individual can vary in terms of orthodontic treatment.

Therefore, adolescents’ own perception toward the severity of their malocclusion is an important contributing factor, formulating their self-esteem levels.^2^ As malocclusion is not a disease, it is defined as the deviation from normal occlusion and it is generally the subjective perception influenced by judgments depending on aesthetic standards of the individual and society.^10^ There is a considerable difference between the clinician and the patient perception toward dental appearance, and both are important for assessing the orthodontic treatment need of an individual. A better understanding of this variation can be evaluated by index of orthodontic treatment need (IOTN), which measures both the normative need and the self-perception need of the individual for orthodontic treatment.

Thus, the purpose of this study was to assess the objective and the subjective orthodontic treatment needs of adolescents and their impact on their self-esteem in Sri Ganganagar city, Rajasthan, India.

## METHOD

The present study was conducted among 10-17-year-old adolescents in a cross-sectional design. It was approved by the institutional ethical review board, and a written consent was taken from the administrators of the selected schools and the students’ guardians for the research.

The required cluster of adolescents was targeted for the children enrolled in various schools. In order to obtain a representative sample, the multi-stage sampling technique was used, for which the city was divided into four different zones in the first stage. Later, four wards were selected randomly from each zone. From each selected ward, a school was chosen based on probability proportional to enrolment size (PPE), making the initial number of selected school to 16. According to PPE, the schools with a high number of regularly attending students were more likely to be selected than schools with low attendance. Out of 16 total schools, two refused to participate, giving an initial school participation rate of 87.5%. To ensure that the sample remained representative of the population, appropriate replacements were made.

Oral health assessment was carried out among 1,784 adolescents from the selected schools. A total of 1,245 students were diagnosed with either one of dental disorders, such as malocclusion, dental trauma, dental caries, and missing teeth. These students were further contacted for the next segment of the study. The selected students who could not obtain the parental consent, who were undergoing orthodontic treatment or were suffering from systemic ailments were excluded from the investigation. Considering the exclusions, the final sample size was 1,140 at a response rate of 91.5%. Thus, 1,140 students were selected for the detailed intraoral examination followed by a questionnaire related to self-esteem. An intraoral examination was performed by two calibrated examiners. WHO type III examination was carried out under natural light using mouth mirrors and sharp probes.^11^

All students were examined for dental health component (DHC) of the IOTN^12^ for assessment of children’s normative treatment need. The aesthetic component (AC) of the IOTN was applied for subjective treatment perception. Students were further examined for dental trauma, tooth loss, and dental caries. For the DHC of IOTN, several traits of malocclusion were assessed: overjet, reverse overjet, overbite, open bite, cross bite, crowding, impeded eruption, defects of cleft lip and palate, as well as any craniofacial anomaly, class II and class III buccal occlusions, and hypodontia. Only the highest scoring trait was used to assess treatment need.

The treatment needs of the patients were categorized into five grades: grades 1 and 2 represent no-or-little need, grade 3 a borderline need, and grades 4 and 5 a definite need for treatment. For the assessment of dental trauma, all maxillary and mandibular anterior teeth from canine to canine was examined for traumatic injury, which was scored using a modified version of Ellis classification.^13^ Tooth loss and untreated decay were further categorized into the number of teeth and zones (masticatory and/or aesthetic). The aesthetic zone was defined as incisors, canines, and first premolars in the upper jaw, and incisors and canines in the lower jaw. The masticatory zone was defined as the second premolars and first and second molars in the upper jaw and both premolars and first and second molars in the lower jaw.^14^ Number and zone (masticatory and/or aesthetic) of the untreated carious lesion and missing teeth were examined using WHO criteria.

The two pre-trained examiners performed intraoral examination among 20-25 adolescents before the main study was carried out, and they were calibrated against a gold standard in the use of the dental disorders, i.e., malocclusion, dental trauma, tooth loss, and untreated carious lesion (intra-examiner and inter-examiner kappa>0.85). The kappa values obtained in the clinical session with respect to intra-examiner and inter-examiner, respectively, were 0.95 and 0.89 for malocclusion, 0.86 and 0.88 for dental trauma, 0.89 and 0.90 for tooth loss, and 0.87 and 0.89 for untreated carious lesions.

After the intraoral examination, the AC of the IOTN has been recorded. It consists of 10 colored photographs with different levels of dental attractiveness, ranked from the most attractive (grade 1) to the least attractive (grade 10). Each adolescent was shown the set of illustrated photographs and was told to compare their dental appearance to these standard photographs and to grade their aesthetics to the nearest resembling photograph. Grading was done as per the score was given by the student. Grades 1-4 represent no-or-little aesthetic need, grades 5-7 borderline aesthetic need, and grades 8-10 definite aesthetic need for orthodontic treatment.

Along with the AC component, the Rosenberg’s Self-Esteem Scale (RSES) proforma was distributed among the students with a prior detailed description of the inventory in regional language for better understanding. The RSES consists of 10 items regarding self-esteem. Each item is rated on a 4-point response scale - 1 is *strongly agree*, and 4 *strongly disagree*. Five items are positively worded (items 1, 3, 4, 7, 10), and five are negatively worded (items 2, 5, 6, 8, 9). The scores for the positively worded items were in the analysis inversed, so that a score of 1 (*strongly agree*) was set to 4. The addition of the item scores gave an overall score from 10-40, with the higher score indicating higher self-esteem.

The descriptive and inferential analysis of the data was done by using IBM SPSS Statistics Windows, version 20.0 (IBM Corp., Armonk, New York, United States). Linear regression analysis was executed to test the individual association of different independent clinical variables with self-esteem scores. The effect of each independent variable was assessed adjusting for that of all others in the model.

## RESULTS

Table 1 depicts the distribution of descriptive and clinical characteristics of 1,140 subjects with a mean age of 15 years old. Mean RSES score among adolescent subjects was found to be 27.1, whereas mean RSES score in males and females was found to be 25.2 and 29.0, respectively.

**Table 1:**
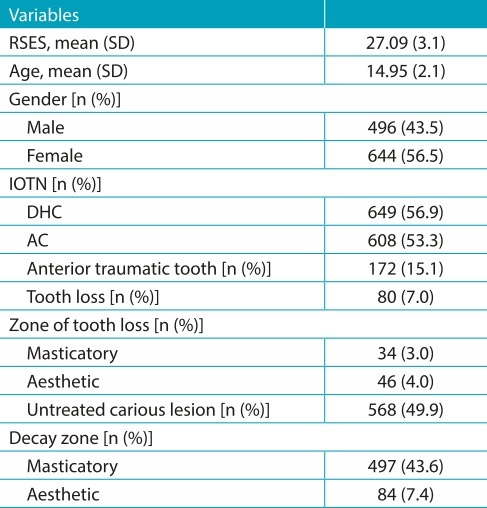
Descriptive and clinical variables of the subjects.

RSES: Rosenberg’s Self-Esteem Scale; SD: standard deviation; IOTN: index of orthodontic treatment need; DHC: dental health component; AC: aesthetic component.

While evaluating malocclusion through IOTN DHC and AC need for treatment, it was found that normative treatment was required by 649 individuals and the subjective perceptions for treatment was found in 608, respectively. A total of 172 individuals were reported with trauma in their anterior teeth, and tooth loss was experienced by 80 individual, while untreated carious lesions were found in 568 individuals.

Table 2 exposes the results of normative treatment need, and subjective perception of treatment need was measured using the DHC and AC of the IOTN in males and females. It was found that normative treatment was required by 17.6% of males and 39.3% of females. Out of which, definite treatment was required by 1.2% of males and 1.4% of females, whereas borderline needs were seen in 3.9% of the males and 6.0% of females. The subjective perception of treatment need by adolescents was low as compared to normative treatment need. In total, 17.2% of males and 36.1% of females considered themselves as needy for the treatment, out of which 1.6% of males and 2.5% of females had the perception of really needing the treatment, whereas subjective perception for borderline need was found in 1.8% of males and 1.5% of females. Females were more concerned and aware about their aesthetic needs, compared to males.

**Table 2:**
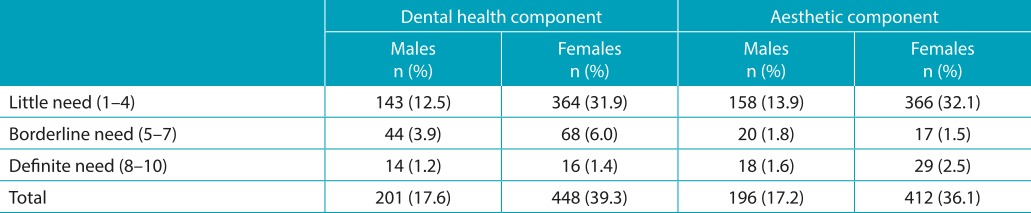
Frequency and prevalence (%) of index of orthodontic treatment need (IOTN) dental health component (DHC) and aesthetic component (AC) scores.

Table 3 displays the stepwise multiple linear regression analysis, which was executed to estimate the linear relationship between RSES (dependent variable) and various independent variables. This analysis revealed that the best predictors in the descending order were DHC, AC, decay (aesthetic zone), decay (masticatory), tooth loss (aesthetic zone), tooth loss (masticatory), and anterior fracture of tooth. IOTN DHC level explained 40.1% of the variance in the model and the cumulative variance provided by all the predictors [DHC, AC, decay (aesthetic zone), decay (masticatory), tooth loss (aesthetic zone), tooth loss (masticatory), anterior fracture of the tooth] was 78%. The dependent variable (RSES) showed the greatest association with model 1, model 3, and model 5, whereas it showed the least association with model 2.

**Table 3:**
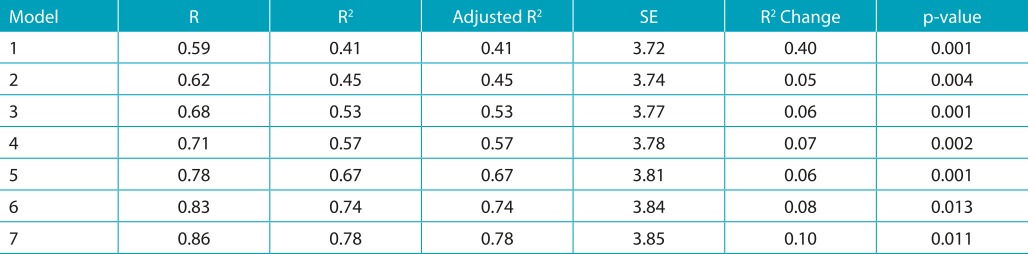
Multiple linear regression model for Rosenberg’s Self-Esteem Scale.

1: Dental health component (DHC); 2: DHC, aesthetic component (AC); 3: DHC, AC, decay (aesthetic zone); 4: DHC, AC, decay (aesthetic zone), decay (masticatory zone); 5: DHC, AC, decay (aesthetic zone), decay (masticatory zone), tooth loss (aesthetic zone); 6: DHC, AC, decay (aesthetic zone), decay (masticatory zone), tooth loss (aesthetic zone), tooth loss (masticatory zone); 7: DHC, AC, decay (aesthetic zone), decay (masticatory zone), tooth loss (aesthetic zone), tooth loss (masticatory zone), anterior fracture of tooth; R: correlation coefficient between the observed and predicted values, in which smaller value indicates that there is little or no linear relationship between the dependent variable and the independent variable; R^2^: a statistical measure of how close the data are to the fitted regression line, also known as the coefficient of determination, or the coefficient of multiple determinations for multiple regression; SE: standard error.

## DISCUSSION

Self-esteem refers to a person’s general sense of worth or acceptance. It has become a household word. Teachers, parents, therapists, and others have focused efforts on boosting self-esteem, as high self-esteem will cause many positive outcomes and benefits. People high in self-esteem claim to be more likable and attractive, to have better relationships, and to make better impressions on others than people with low self-esteem.^8^

Adolescence is a stage of life that offers the potential to prevent from both current impairment and future illness and promoting successful development into productive adulthood. Thus, the purpose of this study was to assess objective and subjective orthodontic treatment needs of adolescents and other dental disorders on the self-esteem of adolescents using RSES. That scale is widely used the self-reported instrument for evaluating individual self-esteem.

It was seen in the present study, according to the multivariate analyses, that, out of all dental disorders, DHC has maximum impact on the adolescents’ self-esteem, followed by AC, which discriminates between the impact of other dental disorders and dental aesthetics on self-esteem. Dental aesthetics plays a vital role in adolescents’ life, affecting their self-esteem level. There was a significant association between self-esteem and perceived dental aesthetics in our investigation, reported similar to Badran^7^ and Kenealy et al’s^15^ studies, which found that subjects who perceive their teeth as less attractive tend to have a lower self-esteem. On the other hand, Sheikh, Mathew and Siew^16^ did not support any association between malocclusion and self-esteem. This could be due to the fact that minor irregularities may be very disturbing for some people, while severe malocclusions may not be of any concern for others.

A study conducted by Dogan et al.^17^ showed a huge difference between IOTN-DHC and IOTN-AC, but the present research found minor differences between both components of IOTN. The difference in findings might be attributed to differences in the studied subjects’ age, as well as cultural differences. The minor differences between IOTN-DHC and IOTN-AC in the present study can be interpreted as a little difference between normative definite treatment need and self-perceived treatment need of the population. It implies that the adolescents enrolled in the present study were more aware and concerned toward their aesthetics and were in constant need to improve their dental appearance and self-esteem.

The present study showed that gender played an important role in the association between self-esteem and malocclusion. The higher RSES scores were found in females. This result is not in agreement with Birkeland, Boe and Wisth,^18^ and the difference might be because, when assessing the attractiveness, females placed themselves at the more attractive end of the scale compared to males, which is in line with another study conducted by Abu Alhaija, Al-Nimri and Al-Khateeb.^19^ However, according to Galambos, Baker and Krahn, attractiveness toward aesthetics changes with age and is not being static for any gender predisposition.^20^

The results of this study suggest that the role of attractiveness in the formation of self-esteem may have to be re-evaluated and refrained. Perhaps adolescent self-esteem is more related to interpersonal performance. Given this framework for understanding adolescent self-concept, demonstrable treatment effects would depend on treatment-related changes in self-protective strategies and social interaction outcomes. In addition, these results call into question the common rationale for providing orthodontic treatment, at least for individuals with mild-to-moderate malocclusion. The full context of adolescent social development needs to be considered in decisions related to orthodontic treatment for young people.

However, the cross-sectional design of the present investigation prevents establishing any causal relationship between dental disorders and the poor self-perception of oral aesthetics, making it impossible to determine whether the associations found preceded or followed the occurrence of the outcome.

In conclusion, the results of the present study indicated that DHC, as well as AC, has a strong association with self-esteem among adolescents, although DHC association was on a little higher side as compared to AC. Based on these findings, the psychosocial problems of an unattractive dental appearance should not be overlooked. Moreover, implementing aesthetic self-evaluation methods may be a useful tool to consider when prioritizing orthodontic treatment modalities.
